# A Rare Case of Melanotic Schwannoma: Utility of Susceptibility Weighted Sequences in Preoperative Imaging

**DOI:** 10.7759/cureus.3068

**Published:** 2018-07-30

**Authors:** Fatima Mubarak, Asra Tanwir, Waseem M Nizamani

**Affiliations:** 1 Radiology, The Aga Khan University, Karachi, PAK; 2 Neurosurgery, Aga Khan University, Karachi, PAK; 3 Neuroradiology, Prince Sultan Military Medical City, Riyadh, SAU

**Keywords:** trigeminal, melanotic, schwannoma, cerebellopontine angle

## Abstract

Intracranial schwannomas account for 8% of all intracranial tumors, out of which 90% are acoustic schwannomas. Other rare varieties include trigeminal melanotic schwannomas that account for 0.2% of all intracranial tumors. Melanotic schwannomas are intracranial tumors that are heavily pigmented due to the presence of melanin. The most common origination of the tumor involves being confined to Meckel’s cave, presenting with features of trigeminal neuralgia, neurasthenia, and numbness. We report a case of a 48-year-old male presenting with dysarthria, left-sided hemiparesis, dysphagia, and headache for the past six months. Magnetic resonance imaging (MRI) confirmed a mass in the right cerebellopontine (CP) angle, which extended into the middle cranial fossa. Our case is interesting because it is the fourth case reported worldwide.

## Introduction

Schwannomas originate from Schwann cells, which normally provide insulation to the myelin sheath covering the peripheral nerves, and they are classified as benign tumors [1]. Facial pain is the most prominent presentation of trigeminal nerve involvement. International literature reports 105 cases of melanotic schwannomas, out of which three cases originated from the trigeminal nerve root [2-3].

## Case presentation

**Figure 1:**
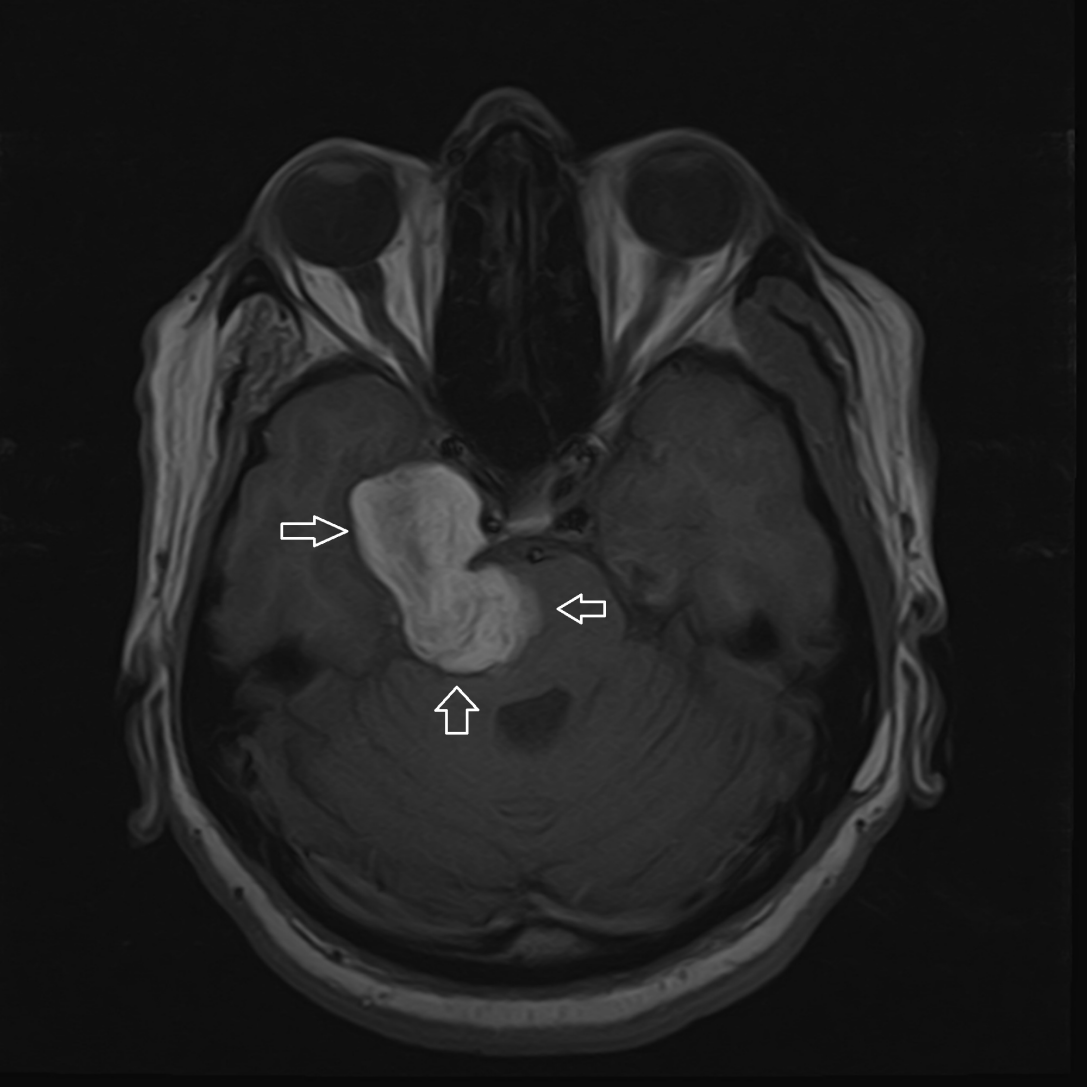
MRI T1 axial brain non-contrast T1 hyperintense lobulated extra-axial mass based on middle and posterior cranial fossae

**Figure 2:**
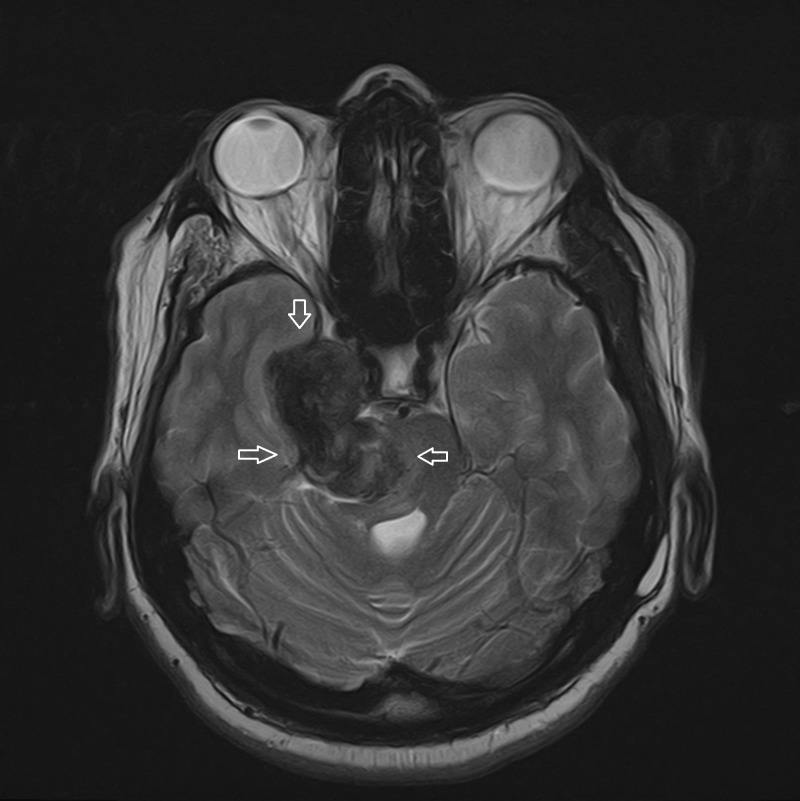
MRI T2 axial brain Significantly hypointense lesion appears to have calcified/hemorrhagic/melanotic internal contents

**Figure 3:**
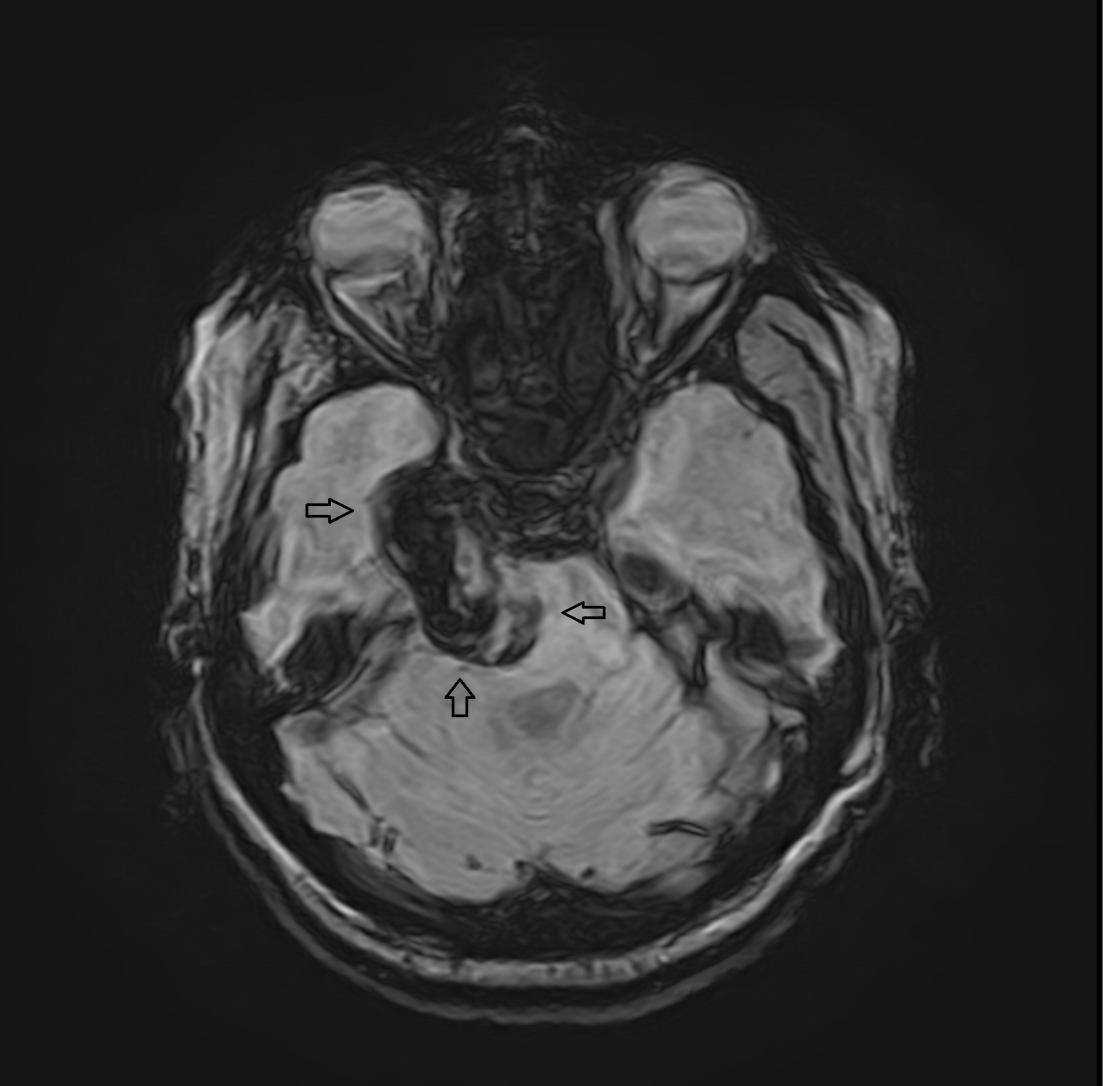
Susceptibility weighted image (SWI) The complete lesion shows a blooming artifact/susceptibility again representing hemorrhagic/calcified/melanotic contents

A 48-year-old male patient presented with dysarthria, left-sided hemiparesis, right-sided facial paresis, dysphagia, difficulty walking, and headache for the past six months. On examination, he was well-oriented to his surroundings with the impairment of the sixth cranial nerve, i.e., uvula deviation to the left, tongue deviation to the left, plantar reflex upgoing, and positive Romberg sign. Magnetic resonance imaging scans of the brain revealed a lobulated lesion in the right cerebellopontine (CP) angle extending into the middle cranial fossa, causing compression of the cavernous sinus and brainstem. The tumor was also abutting the right internal carotid artery medially. The lesion was hyperintense on the T1-weighted (T1W) sequence (Figure 1), hypointense on T2W (Figure 2), and showed abnormal signal dropout on susceptibility weighted imaging (SWI) representing hemorrhage/calcification (Figure 3). We performed a right subtemporal craniotomy and zygomatic osteotomy and resection of the pontotemporal space-occupying lesion. Intraoperative findings include a black-colored tumor arising from the pons and invading the temporal bone.

## Discussion

Schwannomas more frequently occur in the fifth and sixth decades of life with no sex predilection [1]. There are only a few cases of schwannomas reported in the literature with the involvement of the trigeminal nerve, and they have a slight female predilection as compared to a male predilection [4]. A recently reported study revealed that due to the presence of melanin granules, melanotic schwannoma (MS) and metastatic schwannomas have a similar appearance on magnetic resonance imaging (MRI). A dumbbell-shaped growth and cystic structure are the indirect signs that favor MS over metastatic schwannomas [2]. Neuroimaging plays an important role in the diagnosis of trigeminal melanotic schwannoma; MRI being the gold standard in the assessment of tumors. Tumors are isointense or slightly hyperintense on T1-weighted images and have high-signal intensity on T2-weighted images [5]. In our case, the MRI scan showed a lobulated mass in the right cerebellopontine angle, extending into the middle cranial fossa and causing a compression of the cavernous sinus and brainstem, abutting the right internal carotid artery medially, showing subtle enhancement of the internal auditory canal, and abnormal signal dropout on SWI, representing hemorrhage/calcification. The differential possibilities of trigeminal schwannoma with hemorrhage and meningioma were given on the MRI. However, histopathology revealed multiple fragments of a neoplastic lesion arranged in sheets and show extensive melanin pigmentation masking the background morphology of the neoplastic cells. The melanotic trigeminal schwannomas diagnosis is confirmed by the histological examination. A previous study reported that neurosurgeons should be well-informed about the presentation of the Carney complex, which majorly affects the management of the trigeminal melanotic schwannoma pre- and postoperatively. It was also established that the diagnosis cannot be ruled out when psammoma bodies are not present on histological examination [6].

## Conclusions

The use of susceptibility weighted imaging (SWI) for the evaluation of extra-axial lesions was emphasized. A melanotic schwannoma in the trigeminal nerve is rare, and its suspicion can be raised if MRI sequences are carefully read with susceptibility weighted sequences.
